# Measles and rubella seropositivity among suspected measles cases in northwestern region of Nigeria

**DOI:** 10.4102/jphia.v17i1.1354

**Published:** 2026-06-09

**Authors:** Mohammed I. Tahir, Oluwafemi T. Ige, Abdurrahman E. Ahmad, Zainab L. Tanko, Elisha Peter, Zakari Jibrin, Fatima A. Chiroma, Georgina N. Odaibo, Maryam Aminu

**Affiliations:** 1 Department of Medical Laboratory Science, Faculty of Allied Health Sciences, Ahmadu Bello University, Zaria, Nigeria; 2 Department of Medical Microbiology and Parasitology, Kaduna State University, Kaduna, Nigeria; 3 Department of Molecular Laboratory, Yusuf Dantsoho Memorial Hospital, Kaduna, Nigeria; 4 Department of Virology, University of Ibadan, Ibadan, Nigeria; 5 Department of Microbiology, Faculty of Life Sciences, Ahmadu Bello University, Zaria, Nigeria

**Keywords:** measles, rubella, Nigeria, public health, vaccination

## Abstract

**Background:**

Measles is a highly contagious and vaccine-preventable viral disease that results from infection with measles virus. The clinical picture of measles may resemble rubella and other viral exanthems (fever-rash disease).

**Aim:**

This study aimed to determine the measles seropositivity rate among suspected measles cases and evidence of rubella among measles seronegative cases in the seven states of northwestern Nigeria.

**Setting:**

The data were generated from the Medical Laboratory of Yusuf Dantsoho Memorial Hospital Kaduna. The laboratory receives samples from Disease Notification and Surveillance officers of local governments of the seven states of northwestern Nigerian.

**Methods:**

The study was cross-sectional where secondary data of measles case-based surveillance were analysed. The study population comprised of individuals with suspected measles infection living in the seven states of northwestern Nigerian.

**Results:**

Out of 3090 samples tested for measles immunoglobulin M (IgM) antibodies, 1553 (50.3%) tested positive, while 1537 (49.7%) tested negative. Among the negative cases, 1521 were further tested for rubella IgM antibodies, with 157 (10.3%) testing positive.

**Conclusion:**

This high measles seropositivity is concerning considering the ease of access to measles vaccines available at every primary, secondary and even tertiary health facilities spread across northwestern Nigeria.

**Contribution:**

This study reveals that measles virus circulates with high endemicity in the region, with 50.3% serologically confirmed cases among samples from persons suspected to have measles sent for testing. Additionally, 10.3% of individuals suspected having measles but tested negative were found to test positive for rubella.

## Introduction

Measles is a highly contagious viral disease that results from infection with measles virus. The measles virus is a single-stranded ribonucleic acid (RNA) virus of the genus Morbillivirus and the family Paramyxoviridae. The primary means of measles transmission is droplet and aerosols via respiratory route. The symptoms include fever, cough, coryza and conjunctivitis followed by a maculopapular rash starting from the face and moving down to become generalised. Measles can affect most organ systems, pneumonia being the most common complication. Significant morbidity and mortality may follow measles infection.^1^ Even with the efforts to improve surveillance and vaccination coverage such as Expanded Programme on Immunisation (EPI), Integrated Disease Surveillance and Response (IDSR) and community engagement and health education, measles virus continues to cause outbreaks in both low-income and high-income countries.^1^ In 2017, measles was responsible for more than 128 000 deaths globally which decreased from more than 2 million deaths annually before the introduction and widespread use of measles vaccine.^1^

The incidence is highest in late winter and early spring in temperate areas.^2^ Between January 2019 and May 2019, widespread measles outbreaks occurred across all 36 states of Nigeria, including the Federal Capital Territory. By early May, over 28 000 suspected cases had been reported, along with 89 measles-related deaths.^3^ In Nigeria, in a 5-year (2012–2016) review of case-based surveillance data, measles cases increased from February each year, reached a peak in March, then gradually decreased to the lowest level in June, which was then maintained throughout the year.^4^ Certain populations are at a higher risk of disease. For instance, epidemics continue to spread through close contact with among unvaccinated persons, usually in low resourced nations lacking widespread vaccination campaigns. Measles is thought of as a childhood disease, but demographics have shifted to include adults, as more children are immune after vaccination.^2^

Measles virus is highly infectious with R_0_ between 12 and 18 and can cause serious illness with lifelong complication or even death. Prior to the availability of the measles vaccine, more than 90% of children under the age of 15 months could contract the virus.^5^

Rubella infection causes a mild disease among children but can severely affect the foetus if the mother is infected in the early stage of pregnancy. In children, rubella typically causes a mild fever, pink rash starting on the face, swollen lymph nodes behind the ears and sometimes cold-like symptoms such as a runny nose and sore throat. In pregnant mothers, rubella could lead to miscarriage, foetal mortality or the congenital rubella syndrome, a group of debilitating disorders that includes heart problems, blindness, neurological injury and deafness.^6^

Populations achieve and maintain a high level of immunity against these viruses by wide spread vaccination with highly effective and safe measles and rubella vaccines.^6^ Despite this, measles outbreaks continue to occur, particularly among populations with immunity gaps in spite of high overall vaccine coverage, including individuals who received two doses of measles vaccine.^1^

Although the measles incidence has significantly decreased globally as a result of the widespread use of live attenuated vaccines, the virus is still widespread in many impoverished nations with serious outbreaks.^7^ Between 2019 and 2021, the Nigerian government incorporated the second dose of the measles-containing vaccine (MCV2) into the routine immunisation programme. Prior to this, only the first dose of the measles-containing vaccine (MCV1), administered at 9 months of age, was included in the routine schedule.^8^ Thus, measles vaccine is given in two doses: the first at 9 months of age and the second at 15 months.^9^ Despite regular outbreaks and severe measles in Nigeria, many parents are still opposed to vaccinating their children.^10^ Nigeria was reported to have a measles vaccine coverage of only 54% in 2018,^11^ with the lowest coverage of 22.3% for the MCV1 in the North West region. This coverage only increased to 39.1% over a period of 5 years, from 2009 to 2013.^11^ About 85% of reported confirmed cases of measles in Nigeria were from the northern region of the country.^11^

Given the similar clinical presentations of measles and rubella and the combined vaccine products, national and global health leaders have increasingly focused on simultaneous control and management of both diseases.^6^ Laboratory-based surveillance to confirm suspected cases is a crucial part of any measles control programme. A single blood sample obtained as soon as possible following the development of the rash is the basis for routine laboratory confirmation of suspected cases. The test detects measles-specific immunoglobulin M (IgM) in the blood of the suspected case, the IgM persists for 6–8 weeks, thus if seropositive it indicates recent infection.^7^ A measles suspected case is defined as an illness in a patient with fever and generalised maculopapular rash, or in a patient whom a health care worker suspects has measles. A laboratory confirmed case is a suspected case of measles that tested positive to measles virus IgM, and vaccine-associated illness has been ruled out.^12^ Laboratory-based surveillance for measles and rubella is performed throughout the world by the World Health Organization (WHO) Measles and Rubella Laboratory Network (LabNet). The MRLabNet provides for standardised testing and reporting with laboratories serving 166 countries in all WHO regions.^7^

This study was designed to determine the seropositivity rate of measles from samples submitted as part of the measles surveillance from suspected measles cases from the seven states of northwestern Nigeria as well as evidence of rubella virus infection among measles seronegative suspected individuals in the same population.

## Research methods and design

**FIGURE 1:**
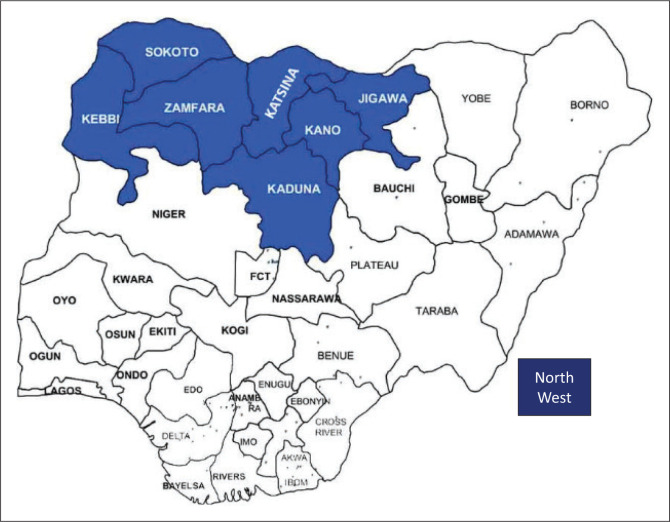
Map of Nigeria highlighting the northwestern region.

The study was cross-sectional where secondary data of a measles case-based surveillance conducted between 07 January 2020 and 08 February 2022 were analysed. The study population comprised of children and adults in the seven states of northwestern Nigeria presenting with a case suspected measles. All of the 3090 measles suspected samples received during the study period from the states were included in the study. Northwestern Nigeria is a geopolitical region that comprises of seven states namely, Jigawa, Kaduna, Katsina, Kano, Kebbi, Sokoto and Zamfara (Figure 1). The region was projected to have over 46 million people in 2019.^13^ Samples of suspected cases were transported from these states to Yusuf Dantsoho Memorial Hospital, Kaduna for measles IgM testing. All measles seronegative samples were tested for rubella IgM. Detection of both measles and rubella IgM was carried out using enzyme linked immunosorbent assay (Euroimmun, Germany) according to manufacturer’s instructions (Order #: EI2610-9601 M; Specificity 98%; Sensitivity 100%).

Basic demographic data of the suspected measles cases were collected from the laboratory database: age, gender, geographical state, results of the measles and rubella IgM. This was analysed using GraphPad Prism (GraphPad Prism v6, GraphPad Software Inc., La Jolla, CA, United States).

### Ethical considerations

Ethical clearance to conduct this study was obtained from Health Research Ethics Committee (HREC) Ministry of Health, Kaduna State, Nigeria (NHREC/17/03/2018).

## Results

Katsina state has the highest, while Kaduna state had the lowest number of samples submitted. The median age of the persons suspected of measles was 4 years (interquartile range: 2–7 years) and children ≤ 5 years had most cases with suspected measles (*n* = 1935; 62.6%) but the lowest percentage of positive measles IgM results (*n* = 904; 46.7%). Males made up 53.5% (*n* = 1652) of the suspected measles cases (Table 1). Of the 3090 samples tested for measles IgM antibodies, 1553 (50.3%) tested positive, while 157 (10.3%) of the 1521 measles seronegative samples tested positive for rubella IgM. There were 16 samples that were negative for measles IgM yet not tested for rubella IgM. Among children 11–15 years with suspected measles, the measles IgM positivity rate was highest compared to the other age groups (*p* = 0.0001) (Table 1) as was the rubella IgM positivity rate compared to the other age groups (*p* = 0.085) (Table 2).

**TABLE 1:** Measles seropositivity rate of suspected measles case according to age group.

Age group (years)	Suspected measles cases		Laboratory-confirmed measles cases	
	*n*	%	*n*	%
≤ 5	1935	62.6	904	46.7
6–10	767	24.8	415	54.1
11–15	237	7.7	145	61.1
16–20	90	2.9	53	58.9
≥ 21	61	2.0	36	59.0

**Total**	**3090**	**100.0**	**1553**	**50.3**

Note: *χ*^2^ = 30.11, degree of freedom (*df*) = 4, *p* = 0.0001.

**TABLE 2:** Rubella seropositivity rate of measles seronegative cases according to age group.

Age group (years)	Measles IgM seronegative cases		Laboratory-confirmed rubella cases	
	*n*	%	*n*	%
≤ 5	1019	67.0	98	9.6
6–10	349	22.9	39	11.2
11–15	91	6.0	14	15.4
16–20	37	2.4	1	2.7
≥ 21	25	1.6	5	20.0

**Total**	**1521**	**100.0**	**157**	**10.3**

Note: *χ*^2^ = 8.191, degree of freedom (*df*) = 4, *p* = 0.085.

IgM, immunoglobulin M.

**FIGURE 2:**
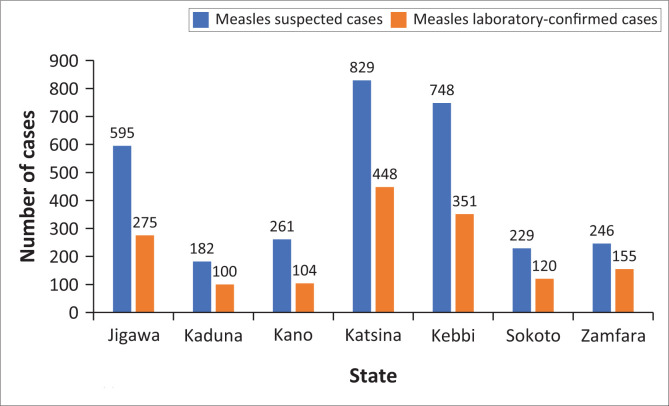
Distribution of suspected measles and laboratory-confirmed cases by States in the Northwestern Region of Nigeria.

Seropositivity of measles IgM was found to be higher (26.4%; *n* = 815/3090) among male suspected measles cases. Rubella IgM positivity was higher among samples from males compared to those from females (5.3%; *n* = 80/157). The frequency of measles suspected cases and laboratory-confirmed cases among the states is presented in Figure 2. Katsina state had the highest number of measles suspected cases, while Kaduna state had the lowest (Figure 2).

**FIGURE 3:**
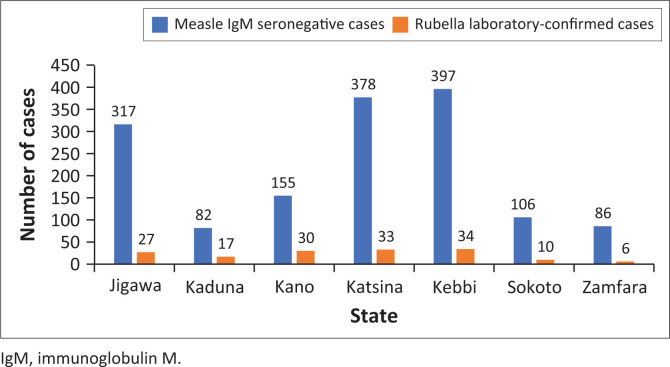
Distribution of laboratory-confirmed rubella virus infection among measles seronegative cases in North Western Nigeria during the study period.

Measles seropositivity rates of each state showed Zamfara state as having the highest seropositivity of 63.0% (*n* = 155/246) and Kano state having the lowest (39.8%; *n* = 104/261) (Table 3). Distribution of seronegative measles suspected cases and laboratory-confirmed cases of rubella virus among the seven states is presented in Figure 3.

**TABLE 3:** Rates of measles IgM seropositivity among suspected measles cases and rubella IgM from suspected measles seronegative individuals.

State	Measles IgM			Rubella IgM		
	Proportion		Seropositivity %	Proportion		Seropositivity %
	*n*	*N*		*n*	*N*	
Jigawa	275	595	46.2	27	317	8.5
Kaduna	100	182	54.9	17	82	20.7
Kano	104	261	39.8	30	155	19.4
Katsina	448	829	54.0	33	378	8.7
Kebbi	351	748	46.9	34	397	8.6
Sokoto	120	229	52.4	10	106	9.4
Zamfara	155	246	63.0	6	86	7.0

**Total**	**1553**	**3090**	**50.3**	**157**	**1521**	**10.3**

*N*, number tested; *n*, number positive; IgM, immunoglobulin M.

Rubella seropositivity among the measles seronegative individuals showed Kaduna state as having the highest seropositivity of 20.7% (*n* = 17/82) (Table 3).

## Discussion

This study was conducted to determine the burden of measles in the region and the distribution of suspected and confirmed measles cases in the northwestern region of Nigeria.

The study found a high burden of measles in the zone. Over half (50.3%) of the samples from suspected measles cases received during the study period were confirmed as measles, indicating a moderately high measles virus infection within the region. This indicates more than 1500 confirmed cases over 2 years for a population of about 3000. The observed measles seropositivity rate exceeding 50% raises important questions: could this reflect outbreaks, suboptimal immunisation coverage and vaccine hesitancy under the EPI, or compromised vaccine potency because of cold chain deficiencies? Disruption of health services, including routine vaccination during the coronavirus disease 2019 pandemic and subsequent prolonged lockdown, lasting for about 75 days in some Nigerian states, may have played a hand in this sustained high seroprevalence rates of measles.

A study of 2019 on the burden of measles in Nigeria revealed that the incidence was higher in the northern states of Nigeria, with Katsina, Sokoto and Yobe, recording more than 200 cases per 100 000 population, while Kebbi and Bauchi recorded between 150 and 199 cases per 100 000 population.^4^ A study among measles suspected cases in three states (Sokoto, Jigawa and Kaduna State) from the north western region of Nigeria in 2016 showed a seroprevalence of measles IgM and immunoglobulin G (IgG) of 35.1% and 86.5%, respectively.^14^

The observed difference in the rate of measles infection between the states may be because of differences in response of residents to mass media immunisation campaigns and routine and supplemental measles vaccination for children.

The high seropositivity of measles found in this study most likely be attributed to low vaccination coverage as the major public health protective measure against measles. During the period 2013–2018, the coverage of the MCV1 in the northwestern region of Nigeria increased only from 22.3% to 39.1%. In contrast, the Southeastern region recorded a higher coverage with rise from 72.2% to 74.8% during the same period.^11^ Data from northern Nigeria showed higher rates of measles infection and lower vaccination coverage than the southern part of the country in a 10-year trend study. Between 2008 and 2018, the incidence rate had showed geographical variation, with higher incidence in the northern part (70.6 per million people) compared to the southern region (17.8 per million).^11^ A review of secondary data from IDSR records of all states in Nigeria over a 5-year period (2012–2016) has also indicated northwestern Nigeria to have the highest incidence of cases (Sokoto: 284.63 cases and Katsina: 246.07 cases per 100 000 population) among the six (*n* = 6) geopolitical regions in Nigeria.^4^ The high burden of measles in northwestern Nigeria found in this study corroborates reports of previous studies and therefore emphasises the need for region-specific interventions to address the high measles incidence.

In this study, children less than 5 years old had the highest number of suspected cases of measles compared to other age groups. This may not be unrelated to the fact that children under the age of 5 years are especially more susceptible to infections with higher incidence when compared with older children and adults with naturally strong immunity. This is because of some factors, which include absence of prior exposure and immunity and an immature immune system that is less able to effectively fight off the measles virus.^12^ Additionally, viral exanthems (rubella, Human Herpesvirus 6 [HHV-6], parvo) could also be mistaken or misdiagnosed as measles. In this study, the age group less than 5 years showed relatively significant low seropositivity of the measles viruses among the age groups. However, higher incidence of measles among children under the age of 5 years were reported in many other studies conducted in Nigeria.^11,15,16,17,18^

The age group of 11–15 years with the highest seropositivity could have been caused by vaccination gaps because of unscheduled changes in vaccine dates such as stockouts, missed opportunities for vaccination or not completing the vaccination series. It indicates a persistent immunity gap, likely resulting from historical lapses in vaccination coverage and possible cold chain failures. It is important to include in the vaccination coverage assessment the vaccination coverage of MCV1 and MCV2. In endemic settings, most individuals are expected to acquire measles immunity by age 15 years, either through vaccination or natural infection. Inadequate vaccination coverage lowers population immunity below the herd immunity threshold, creating cycles of susceptibility and increasing the likelihood of measles outbreaks every 2–5 years unless vaccine uptake and delivery systems are significantly improved.^19^ Immunity from childhood measles vaccination can gradually wane over time. If the initial measles vaccine doses were administered when these individuals were younger, their immunity might be suboptimal as they enter adolescence, making them more susceptible to infection.^20^

This study detected the presence of rubella IgM in samples of the measles seronegative patients, with a seropositivity of 10.3%. This rate obtained is higher than the findings in a study conducted in a northwestern states of Nigeria in 2015 but comparable to the findings in Tanzania with 10.9% in 2014.^9,21^

A review of secondary data from IDSR records of all states in Nigeria over a 5-year period indicated northwestern region to have the highest incidence among the six geopolitical regions of Nigeria.^4^ The northern Nigerian region has recorded higher measles incidence and lower vaccination coverage than the southern part of the country in a 10-year trend study.^11^ Therefore, the high seropositivity reported in our study may be attributed to the low vaccination coverage during the study period in the region.

Most of the 1553 samples that tested positive for measles virus were from children less than 5 years of age (*n* = 904/1553, 58.2%) indicating a high burden of the disease among this age group. However, the highest seropositivity of 61% was found among those 11–15 years of age. This finding is in support of the call for a booster dose of measles vaccine among older children. A research study indicated that, in the absence of exposure to the wild-type virus, approximately 8.9% of individuals experienced a reduction in vaccine-neutralising antibodies to levels below the protective threshold within around 7 years after receiving the vaccine.^18^

Measles vaccine has been in use for nearly 60 years. Evidence shows that it is safe, effective and inexpensive.^12^ The introduction of measles vaccine into routine immunisation programme results in a marked reduction in incidence of the disease and its associated morbidity and mortality. When high levels of vaccine coverage are attained (i.e. WHO recommended vaccine coverage > 95%), measles incidence decreases and the intervals between outbreaks are lengthened.^12^

In this study, Kaduna and Kano states had the highest rubella virus seropositivity of 20.0% and 19.4%, respectively. A similar study conducted by Fatiregun et al.^22^ reported in 2014 in the southwestern part of Nigeria documented an overall seropositivity of 5.4%^22^ which is far lower than the 10.5% obtained in this study.

Most studies conducted on rubella in Nigeria were among pregnant women, and for instance, an IgG seroprevalence of 77% in Lagos^20^ and Ilorin 1.5%^23^ were reported. A study conducted among infants across five African countries: Burkina Faso, Rwanda, Tanzania, Zambia and Zimbabwe revealed an IgM seropositivity of 31.1%.^9^

## Conclusion

The findings of this study reveal a very high measles seropositivity rate across the population, with notably seropositivity among older age groups – highlighting potential immunity gaps and missed opportunities for earlier immunisation. Additionally, the high rubella IgM seropositivity underscores ongoing transmission and the need for broader preventive strategies. To address these challenges, it is imperative to strengthen routine immunisation services, enhance the reach and effectiveness of Supplementary Immunisation Activities, and transition to the combined measles-rubella vaccine to ensure comprehensive protection against both diseases. This study highlighted the presence of significant rubella antibodies among seronegative measles cases. Therefore, parallel serotesting of measles and rubella should be adopted to intensify the detection of rubella antibodies in all suspected cases of measles, thereby improving the management of suspected cases and prevention. Primary healthcare facilities in these states should identify gaps in immunisation programmes so that the targeted group is indeed reached. The need to develop and implement a region-specific strategy to improve the uptake of vaccines in the northwestern states in Nigeria is crucial and urgent. Achieving at least 95% vaccination coverage with two doses of a measles- and rubella-containing vaccine at both national and district levels remains a critical target for measles and rubella elimination, as recommended by the WHO.
